# Hal: an Automated Pipeline for Phylogenetic Analyses of Genomic Data

**DOI:** 10.1371/currents.RRN1213

**Published:** 2011-02-07

**Authors:** Barbara Robbertse, Ryan J. Yoder, Alex Boyd, John Reeves, Joseph W. Spatafora

**Affiliations:** ^*^National Center for Biotechnology Information, Bethesda, Maryland; ^†^Peace Corps; ^‡^Oregon State University and ^§^Bonzi Software Development

## Abstract

The rapid increase in genomic and genome-scale data is resulting in unprecedented levels of discrete sequence data available for phylogenetic analyses. Major analytical impasses exist, however, prior to analyzing these data with existing phylogenetic software. Obstacles include the management of large data sets without standardized naming conventions, identification and filtering of orthologous clusters of proteins or genes, and the assembly of alignments of orthologous sequence data into individual and concatenated super alignments. Here we report the production of an automated pipeline, Hal that produces multiple alignments and trees from genomic data. These alignments can be produced by a choice of four alignment programs and analyzed by a variety of phylogenetic programs. In short, the Hal pipeline connects the programs BLASTP, MCL, user specified alignment programs, GBlocks, ProtTest and user specified phylogenetic programs to produce species trees. The script is available at sourceforge (http://sourceforge.net/projects/bio-hal/). The results from an example analysis of Kingdom Fungi are briefly discussed.

## Introduction        

        As a direct result of genome biology and high throughput sequencing technologies, the bottleneck in producing multi-gene species trees has shifted from generating data to processing and analyzing data. Genome-based projects stand to contribute ample data for large multi-gene phylogenies, but mining and parsing these data manually is impractical and requires automation. This paper describes a command line program (Hal) that brings together a number of bioinformatic applications into an efficient pipeline that inputs unaligned proteins sequences in fasta format and generate species trees from super alignments containing several orthologous protein sequences in a fully automated manner.

        Several available methods exist for automated multi-genome ortholog identification with the strategies for finding orthologs roughly divided into two categories, including graph-based and tree construction methods.  Graph-based methods (e.g., MCL [1], Mult-Paranoid [2] and OrthoMCL [3]) mainly involve parsing of BLAST [4] results while most tree-based methods employ distance-based phylogenetic analysis, such as Orthostrapper [5], SDI tree reconciliation [6], RAP [7], COCCO-CL [8] and LOFT [9].  Some of the tree-based or hybrid methods require a stable species tree, and thus are not suitable when the goal is the estimation of phylogenetic relationships, or are not available as a command line tool but only as a graphical user interface (GUI).  Programs such as Ensembl Compara [10], TreeFam [11] and OrthologID [12] combine both strategies, either using trees to guide the clustering procedure or to refine clusters.  

        As part of the Assembling the Fungal Tree of Life (AFTOL) project we are collecting and analyzing genome-scale data to produce robust phylogenetic hypotheses for deep and problematic nodes within the Kingdom Fungi.  Towards this end we have developed a pipeline of Perl scripts, collectively referred to as Hal, that connect existing programs to automate the following: the mining of conserved orthologous sequences among a designated number of genomes followed by alignment and phylogenetic analysis of these sequences.  The most appropriate model of evolution, for both individual and concatenated super alignments, is determined and sets of concatenated alignments are subjected to a variety of phylogenetic analyses.  Previous and modified versions of this pipeline have already been successfully applied among fungi [13], bacteria [14] [15] [16] and insects [17].  With no manual intervention this pipeline provides a robust and efficient evaluation of higher-level phylogenetic relationships. 

## A Description of the Pipeline

        The Hal pipeline inputs predicted protein sequences in fasta format from sequenced genomes and produces species trees from multi-gene super alignments.  In doing so, Hal executes the following basic steps: orthologous cluster identification, alignment of clusters, alignment editing, alignment concatenation, model testing, and phylogenetic analysis (Fig 1).  Ortholog identification involves all-vs-all BLASTP, protein clustering, cluster selection and filtering.  If a run is disrupted for any reason, the program can be restarted at the beginning of the last uncompleted step.  Intermediate scripts that process each of the individual steps can also be used independently, provided the expected formatted files are provided.  The main log provides useful information if an error occurred and lists the steps that have been reached.  At the start of a run the following options are available: running the program locally or using a Sun Grid Engine (SGE), choice of alignment program, choice of inflation parameters to use with clustering, minimum alignment length allowed, minimum percentage data allowed per cluster, outgroup designation and choice of phylogenetic program.  All the intermediate files can be in the output or only the final products.  Details about the contents of these files are available in the documentation distributed with the program.  Before sequences are submitted to BLASTALL they are first subjected to a quality check.  Any fasta sequences with duplicated headers or sequences containing non-IUPAC characters are removed.  Currently, there are no standards for the header format among different institutions and sequencing centers, which makes it difficult to automatically parse out a unique name for each fasta sequence.  Instead a map is created of the original headers and a short and unique alias name is provided.

**Figure:**
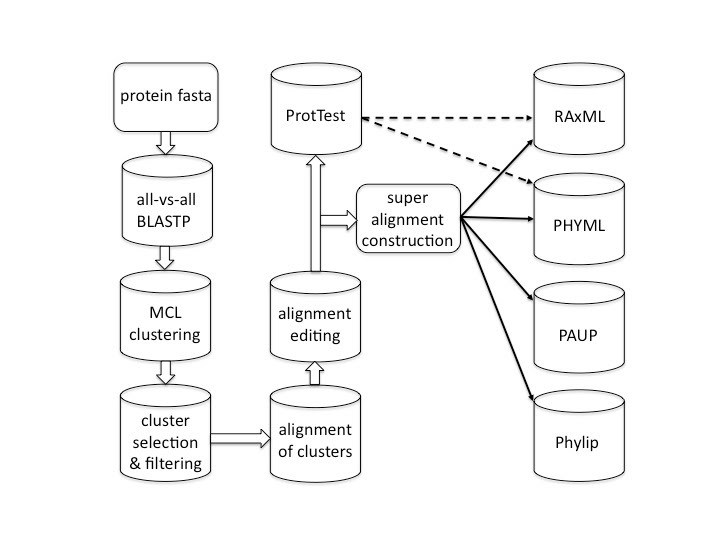
**Fig. 1.**  Flow diagram of the Hal pipeline.

        In the following paragraphs each step is discussed along with its default settings, but more details are provided in the program documentation.  Not all options are currently available for modification when Hal is executed, but if started manually some intermediate scripts have more options available for modification to satisfy user preferences. 

## Ortholog Identification

*All-vs-all BLASTP*. – The all-vs-all BLASP [4] is executed with a cutoff e-value of 1e-1 and set to report BLAST alignments and one-line descriptions for a number of database sequences equal to the number of organisms analyzed.  For example, if 50 genomes are analyzed, the BLAST report is limited to 50 sequences.  The –F option is set to mS which results in soft filtering of low complexity regions and masks segments of low complexity during the search phase of BLAST but not during the alignment phase.  Moreno-Hagelsieb and Latimer [18] studied the effect of masking low-information segments and found that most of the improvement of detecting orthologs as reciprocal best hits can be achieved by soft filtering alone.  BLASTALL default values are used for the rest of the options but can be modified if the wrapper script is started manually. 

*MCL clustering, cluster selection and filtering. – *A wrapper script takes in the raw BLAST files as input and executes the MCL clustering program [1] with the programs default options except for the inflation parameter, which can be set by the user.  MCL creates a similarity matrix from e-values and then clusters the proteins into related groups.  The main parameter that influences the size of a cluster is the inflation parameter.  A lower inflation parameter represents a more lenient clustering parameter (fewer clusters with more proteins/cluster) and a higher inflation parameter represents a more stringent clustering parameter (more clusters with fewer proteins/cluster).  The wrapper script executes the MCL program across a range of inflation parameters with the default range set at 1.1, 1.2, 1.3, 1.4, 1.5, 1.7, 2.0, 2.5, 3.0, 3.5, 4.0, 4.5 and 5.0.  Using a range of settings for the inflation parameter accommodates slow and fast evolving genes, which may provide resolution at different nodes within a phylogenetic tree.  

        After MCL clustering across a range of parameters, only single copy clusters (one protein per organism) are selected and filtered starting from the most stringent inflation parameter so that only non-redundant clusters remain where all proteins have best hits to proteins from its own cluster.  That is, filtered clusters can only be represented once in the final alignment and they can not contain a protein with a best hit to a protein that resides within another cluster.  With increasing number of genomes compared, however, the number of single copy clusters with all genomes represented decreases quickly.  This phenomenon may be due to gene loss in a particular genome, error in ortholog assessment, poorly or incompletely annotated genomic input data or some combination of the above.  To alleviate this problem and maximize the amount of data available for phylogenetic analyses, an additional option is available to allow clusters that contain missing taxa.  This option allows the user to set the minimum allowed percentage of taxa present per cluster.  For example, setting the minimum included per cluster value at 80% will allow a maximum of 20% missing data within a given cluster.**

## Generation of Alignments

*Alignment programs*. – The default alignment program is MUSCLE [19], which is executed with the program’s default settings except the input order of the sequences are kept by specifying –stable.  Other supported alignment programs are PROBCONS (default options applied) [20], MAFFT (default options applied) [21] and CLUSTALW (default options applied) [22]. 

        *Alignment Editing*. – Currently there are no options given to refine the setting in each alignment program.  Rather, alignment optimization involves a choice between the four alignment algorithms and the ability to edit the alignments using a customized Perl script (remgaps) and Gblocks [23] with different settings.  Previous versions of the pipeline aligned only sequences reported in BLASTP to limit comparison of highly divergent regions [13].  However, in the current version of Hal the whole protein is aligned and the program Gblocks is used to assist with the removal of poorly aligned positions and highly divergent regions. Currently there are three levels of alignment parsing that result in three different alignment lengths.  These include: 1) a non-Gblocks script that remove all gap-containing columns (remgaps); 2) Gblocks parsing with conservative settings (maximum number of contiguous nonconserved positions allowed is 4; minimum length of a block allowed is 10); and 3) GBlocks parsing with liberal settings (maximum number of contiguous nonconserved positions allowed is 8; minimum length of a block allowed is 5).  Contiguous nonconserved regions are gap-rich regions, i.e., poorly aligned, and blocks are conserved well aligned regions.  The remgaps option is the most strict and frequently results in a decrease in the total number of orthologous clusters incorporated into the alignment due to the alignment length of a given cluster falling below the designated threshold length.  Gblocks with conservative settings will also result in less amino acid data incorporated into the final analysis but typically has less of an effect than remgaps.  GBlocks with liberal settings allows for the maximum amount of primary sequence data and orthologous clusters to be included in a super alignment.  Table 2 provides an example of the different amounts of data included across a series of analyses for Kingdom Fungi.  The allowance of clusters with missing data does not affect the considering of gap-containing columns, since the alignments are performed on each individual protein cluster and normalization of alignments, i.e., introduction of taxa with missing data into an alignment, is performed after alignment parsing.

*Concatenation of orthologous sequences into super alignments.* **– **Individual alignments that will be used to build the super alignment are filtered by the minimum alignment length allowed as set by the user at the start of the Hal run.  Mapped alias names are exchanged for mapped organism names and all the alignments are normalized so that each alignment has the same taxa in the same order.  Missing data are represented with the question mark character.  Finally, the alignments parsed as described above are concatenated to produce three super alignments (remgaps, Gblocks conservative, Gblocks liberal), each resulting in different lengths and number of allowed gaps.  Depending on the minimum alignment length value set for the individual cluster alignments, the three super alignments may also differ in the number of represented clusters. 

## Phylogenetic Analyses

*Amino acid substitution model testing.* **– **The program ProtTest is used for estimating the best model for each alignment [24].  This is determined using fast optimization according to the AIC model selection strategy by default.  For each alignment, the raw output (directly from ProtTest) produces a table of ranked models under all selections strategies with details about models under the AIC framework for each alignment.  We developed a parsing script that reads a directory of these output files and produces four output files: 1) a tab delimited file that lists the best model for each alignment according to the AIC model selection strategy; 2) a tab delimited file that lists and ranks all the models that did not score entirely 0.00 by different model selection (AIC, AICc, BIC) strategies for each alignment; 3) a tab delimited file that summarizes the frequency by which each model had the highest rank in a set of alignments analyzed; and 4) an alignment partition file that lists positions and models in a format to be used by RAxML.  The most frequently highest ranked model is used when the one model option is selected in the phylogenetic analysis of the super alignment.  

*Phylogenetic programs.* **– **A choice of four phylogenetic programs is supported in Hal, two maximum likelihood algorithms, PhyML [25] and RAxML [26], a maximum parsimony (MP) program (PAUP) [27], and a neighbor joining (NJ) distance analysis using PHYLIP [28].  There is no default tree construction and at least one of these programs must be set as an option to include a phylogenetic analysis as part of the automated Hal run.  There is a wrapper script for each phylogenetic program that populates the options and executes the phylogenetic analysis.  Currently, the minimum user defined variables are available as part of the automated pipeline (e.g., bootstrap and outgroup designation).  By default the MP analysis includes a heuristic search, evaluating 100 random-addition replicates (maximum trees = 100) and excluding uninformative characters.  The NJ analysis includes a distance matrix using the JTT amino acid substitution model with no variation among sites using the Neighbor-Joining method of clustering and construction of a strict majority rule consensus tree.  As mentioned above, the model of evolution for RAxML can be specified by partition or for the entire super alignment, and the model of evolution can be specified for the entire super alignment in PHYML.

        Because Hal automatically produces three super alignments, three trees are produced by each phylogenetic analysis.  In this pipeline, confidence in tree topology is tested not only by the range of phylogenetic algorithms and bootstrap support, but also by the variation in super alignment construction provided by remgaps, Gblocks conservative and Gblocks liberal.  Additional super alignment constructions may be performed by restarting a Hal analysis with another alignment program specified; in this case, the pipeline will automatically start with alignment of individual clusters (Fig 1.). 

## Implementation

        Hal is a Perl command line program, meaning there is no GUI.  It was developed on a 64-bit Linux architecture, currently running Red Hat Linux 3.2.3, Linux version 2.4.2.1.  However, Hal should be able to be run in any Linux environment.  It is most efficient when using a SGE, which significantly decreases processing time since serial jobs are run on several processors.  Hal can also run on a single machine, but this will take considerably more time depending on the number of taxa and size of the input genomes.  Running Hal on a 32-bit machine may present a problem of insufficient memory for larger analyses.  Included in the Hal distribution is a script, which checks that all the dependencies of Hal are met, helping you to determine what programs are currently on your system.  Hal needs a fairly recent version of Perl, a few standard Perl modules, BioPerl formatdb, BLASTALL, a supported alignment program, Gblocks, a supported phylogenetic program and ProtTest (depending on the choice of phylogenetic analysis).  More details such as program versions are available in the install document.

## Results of a Hal Analysis of Kingdom Fungi

        Kingdom Fungi currently has more genomes sequenced than any other eukaryote kingdom and the availability of these data is greatly advancing investigations into evolutionary relationships of the Fungal Tree of Life [13] [29] [30] [31] [32] [33] [34].  As an example of the Hal pipeline, we analyzed 52 genomes (Appendix 1) from the Kingdom Fungi with and without *Drosophila melanogaster* as an outgroup.  These genomes represented 8 of the 15 subphyla/phyla currently recognized in the most recent classification of the Fungi (Hibbett et al. 2007) and served as a core dataset for development of Hal.  Analyses were performed across a range of 60, 80 and 100% minimum included taxa per cluster, clusters were aligned using MUSCLE with the most likely model of amino acid substitution assigned to each protein partition of the super alignment, and phylogenetic analyses were conducted in RAxML.  In Figure 2 the topology and bootstrap support of the most likely tree, from an analysis with minimum 80% taxa present and Gblocks liberal alignment, is compared with eight other analyses.  Table 1 provides a summary of cluster statistics for each alignment across all analyses.

**Table 1.**  Summary statistics for Hal analyses of 52 fungal genomes. Alignments were produced with and without D. melanogaster included as an outgroup. 

**Table:** 

	100% genomes/cluster	80% genomes/cluster	60% genomes/cluster
Alignments	# clusters/ #aa/ model^4^	# clusters/ #aa/ model^4^	# clusters/ #aa/ model^4^
with *D. melanogaster *			
Gblocks lib.^1^	20/ 5053/ RtREV+IGF	161/ 24608/ RtREV+IGF	373/ 48233/ RtREV+IGF
Gblocks con.^2^	20/ 3946/ RtREV+IGF	156/ 18886/ RtREV+IGF	361/ 35762/ RtREV+IGF
RemGaps^3^	20/ 3312/ RtREV+IGF	156/ 16935/ RtREV+IGF	380/ 37542/ WAG+IG
w/o *D. melanogaster*			
Gblocks lib.^1^	34/ 398/ RtREV+IGF	210/ 32849/ RtREV+IGF	406/ 56728/ RtREV+IGF
Gblock con.^2^	33/ 6680/ RtREV+IGF	202/ 25148/ RtREV+IGF	389/ 42414/ RtREV+IGF
RemGaps^3^	32/ 5950/ RtREV+IGF	199/ 23031/ RtREV+IGF	388/ 42163/ RtREV+IGF

^1^Alignments edited using Gblocks liberal settings.

^2^Alignments edited using Gblocks conservative settings.

^3^Alignments edited using RemGaps.

^4^Most common amino acid substitution model.

**Figure:**
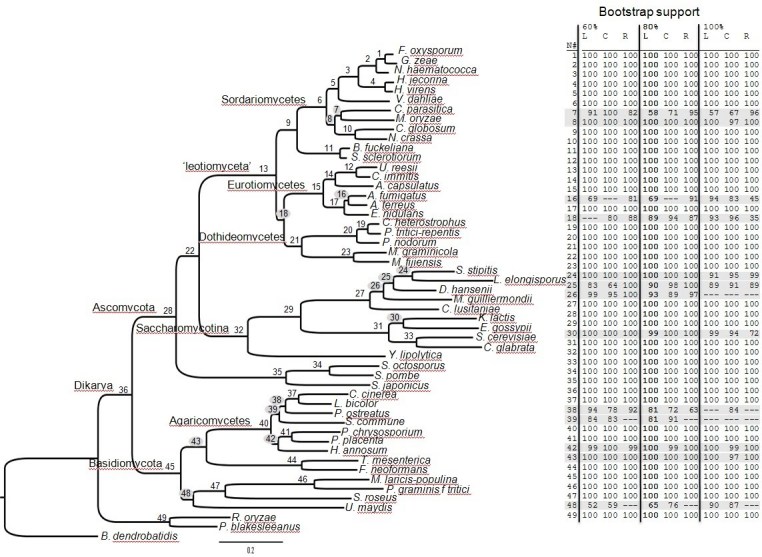
**Fig. 2**.  A phylgenetic tree from a RAxML analysis including at least 80% of the taxa per protein cluster in a concatenated MUSCLE alignment adjusted by Gblocks with liberal settings.  Nodes are numbered (N#) 1 to 49 and in the associated table corresponding bootstrap partitions (BP) are provided from analyses of alignments parsed with remgaps (R), Gblocks with conservative settings (C), and Gblocks with liberal settings (L), where at least 60%, 80% and 100% of the taxa are present per protein cluster.  Nodes highlighted in gray indicate BP below 100% or no support (---) in one or more analyses.  See Table 1 for number of proteins and amino acid positions in each analysis.

        These fully automated Hal analyses recovered all currently accepted higher-level phylogenetic relationships (e.g., monophyly of Sordariomycetes, Ascomycota, Agaricomycotina, Basidiomycota, Dikarya, etc.) within the Fungi (Fig. 2; [35] [36]).  Eight out of the 49 internal nodes of the tree (nodes 7, 16, 18, 25, 26, 38, 39, 48), however, were either resolved differently across the datasets or were characterized by varying measures of support.  Four of these eight nodes (nodes 7, 26, 38, 39) were characterized by higher bootstrap partitions (BP) with an increase in the amount of data, while the remaining four nodes were characterized by somewhat inconsistent measures of support.  The utility and interpretation of BP in phylogenomic analyses that involve numerous concatenated genes or proteins has been questioned [37].  Examples exist where nodes that are well supported in one analysis are resolved differently in another, or individual nodes are characterized by highly variable BP values (e.g., Taprhinomycotina in [38]).  Several reasons have been proposed to explain this phenomenon including unequal rates of evolution, compositional biases, conflict among data partitions, deep coalescences and insufficient taxon sampling [37] [39] [40].  Although the Hal pipeline does not specifically address these potential shortcomings (but see below), varying the analyses as a function of allowable missing data and therefore ortholog inclusion, does provide a heuristic assessment of which nodes may be characterized by inconsistent descriptors of support and in need of additional analyses (Fig. 2). 

## Future Development

        Significant improvements have been made to the Hal pipeline since its first implementation (Robbertse et al. 2006), resulting in a faster and fully automated pipeline with more user options.  We are continuing with further developments that include:

- Expansion of user-defined options for alignment and phylogenetic programs.
- Inclusion of additional ortholog identification methods (e.g., OrthoMCL, Multiparanoid).
- Inclusion of taxa for which only transcriptome data (e.g., ESTs) exist.
- Inclusion of conflict analyses for detection of incongruent phylogenetic signals among orthologous clusters.
- Inclusion of phylogenetic network algorithms (e.g., SplitTrees4 [41]).
- A result summary of multiple analyses (e.g., Figure 2).
- Inclusion of nucleotide sequence data.

## Funding

        This research was supported by a National Science Foundation grant (DEB-0732993) to J.W.S.  Any opinions, findings, and conclusions or recommendations expressed in this material are those of the authors and do not necessarily reflect the views of the National Science Foundation.

## Acknowledgments

        We thank the Center for Genome Research and Biocomputing at Oregon State University for providing computational resources and Chris Sullivan for assistance implementing Hal in SGE.  We also thank the principal investigators, genome institutes/consortia (Broad Institute of Harvard and MIT, US Department of Energy Joint Genome Institute, Sanger Institute and Génolevures consortium) and the respective funding agencies associated, for the availability of genomic sequences.  

## Competing Interests

The authors have declared that no competing interests exist.

## Appendix  

### Taxon sampling for Hal analysis of Kingdom Fungi.

**Table:** 

**Organism**	**PHYLUM/SUBPHYLUM**	**CLASS**	**DATABASES/SOURCE**
*Drosophila melanogaster*	Arthropoda	Insecta	FlyBase
*Phycomyces blakesleeanus*	Mucoromycotina	Mucoromycetes	DOE Joint Genome Institute
*Rhizopus oryzae*	Mucoromycotina	Mucoromycetes	Broad Institute
*Batrachochytrium dendrobatidis*	Chytridiomycota	Chytridiomycetes	Broad Institute
*Hypocrea virens*	Ascomycota	Sordariomycetes	DOE Joint Genome Institute
*Fusarium oxysporum lycopersici*	Ascomycota	Sordariomycetes	Broad Institute
*Nectria haematococca*	Ascomycota	Sordariomycetes	DOE Joint Genome Institute
*Hypocrea jecorina*	Ascomycota	Sordariomycetes	North Carolina State Univ
*Verticillium dahliae*	Ascomycota	Sordariomycetes	Broad Institute
*Chaetomium globosum*	Ascomycota	Sordariomycetes	Broad Institute
*Neurospora crassa*	Ascomycota	Sordariomycetes	Broad Institute
*Magnaporthe oryzae*	Ascomycota	Sordariomycetes	International Rice Blast Genome Consortium
*Coprinus cinereus*	Basidiomycota	Agaricomycetes	Broad Institute
*Laccaria bicolor*	Basidiomycota	Agaricomycetes	Institut National de la Recherche Agronomique
*Pleurotus ostreatus*	Basidiomycota	Agaricomycetes	DOE Joint Genome Institute
*Schizophyllum commune*	Basidiomycota	Agaricomycetes	DOE Joint Genome Institute
*Heterobasidion annosum*	Basidiomycota	Agaricomycetes	DOE Joint Genome Institute
*Phanerochaete chrysosporium*	Basidiomycota	Agaricomycetes	DOE Joint Genome Institute
*Postia placenta*	Basidiomycota	Agaricomycetes	DOE Joint Genome Institute
*Cryptococcus neoformans var. grubii*	Basidiomycota	Tremellomycetes	Duke Univ, Broad Institute
*Tremella mesenterica*	Basidiomycota	Tremellomycetes	DOE Joint Genome Institute
*Ustilago maydis*	Basidiomycota	Ustilaginomycetes	Exelixis Inc, Broad Institute
*Melampsora laricis-populina*	Basidiomycota	Pucciniomycetes	DOE Joint Genome Institute
*Puccinia graminis tritici*	Basidiomycota	Pucciniomycetes	Univ of Minnesota, Broad Institute
*Sporobolomyces roseus*	Basidiomycota	Pucciniomycetes	Trinity College, Dublin, DOE Joint Genome Institute
*Cryphonectria parasitica*	Ascomycota	Sordariomycetes	DOE Joint Genome Institute
*Schizosaccharomyces octosporus*	Ascomycota	Schizosaccharomycetes	Broad Institute
*Schizosaccharomyces japonicus*	Ascomycota	Schizosaccharomycetes	Univ of Massachusetts, Broad Institute
*Schizosaccharomyces pombe*	Ascomycota	Schizosaccharomycetes	Schizosaccharomyces pombe Genome Sequencing Consortium
*Clavispora lusitaniae*	Ascomycota	Saccharomycetes	Broad Institute
*Meyerozyma guilliermondii*	Ascomycota	Saccharomycetes	Broad Institute
*Lodderomyces elongisporus*	Ascomycota	Saccharomycetes	Broad Institute
*Saccharomyces cerevisiae*	Ascomycota	Saccharomycetes	Stanford Univ
*Scheffersomyces stipitis*	Ascomycota	Saccharomycetes	Boston College
*Debaryomyces hansenii*	Ascomycota	Saccharomycetes	Génolevures Consortium
*Eremothecium gossypii*	Ascomycota	Saccharomycetes	Biozentrum, University of Basel, Switzerland
*Kluyveromyces lactis*	Ascomycota	Saccharomycetes	CNRS, France
*Candida glabrata*	Ascomycota	Saccharomycetes	Génolevures Consortium
*Yarrowia lipolytica*	Ascomycota	Saccharomycetes	Génolevures Consortium
*Botryotinia fuckeliana*	Ascomycota	Leotiomycetes	Syngenta AG, Broad Institute
*Sclerotinia sclerotiorum*	Ascomycota	Leotiomycetes	Broad Institute
*Ajellomyces capsulatus*	Ascomycota	Eurotiomycetes	Broad Institute
*Uncinocarpus reesii*	Ascomycota	Eurotiomycetes	Broad Institute
*Mycosphaerella graminicola*	Ascomycota	Dothideomycetes	DOE Joint Genome Institute, USDA-ARS, Purdue Univ
*Mycosphaerella fijiensis*	Ascomycota	Dothideomycetes	DOE Joint Genome Institute, USDA-ARS, Purdue Univ
*Stagonospora nodorum*	Ascomycota	Dothideomycetes	Broad Institute
*Pyrenophora tritici-repentis*	Ascomycota	Dothideomycetes	Broad Institute
*Cochliobolus heterostrophus*	Ascomycota	Dothideomycetes	DOE Joint Genome Institute, USDA-ARS, Purdue Univ
*Aspergillus fumigatus*	Ascomycota	Eurotiomycetes	Sanger, J. Craig Venter Institute
*Aspergillus nidulans*	Ascomycota	Eurotiomycetes	Broad Institute
*Aspergillus terreus*	Ascomycota	Eurotiomycetes	Broad Institute, J. Craig Venter Institute, The University of Manchester, UK
